# Degenerative joint disease in the temporomandibular joint with fibrous ankylosis in a rhesus macaque (*Macaca mulatta*)

**DOI:** 10.1186/s42826-020-00052-2

**Published:** 2020-07-02

**Authors:** Su-Mi Kim, Chang-Gok Woo, Jong-Min Kim

**Affiliations:** 1 Seoul National University Dental Hospital, 103 Daehak-ro Jongno-gu, Seoul, 110-799 Korea; 2 Department of Pathology, Chungbuk National University College of Medicine, Cheongju, 361-763 Korea; 3 Xenotransplantation Research Center, 103 Daehar-ro Jongno-gu, Seoul, 110-799 Korea; 4 Institute of Endemic Diseases, 103 Daehar-ro Jongno-gu, Seoul, 110-799 Korea; 5 Cancer Research Institute, Seoul National University College of Medicine, 103 Daehak-ro Jongno-gu, Seoul, 110-799 Korea

**Keywords:** Fibrous ankylosis, Degenerative joint disease, Computed tomography, Temporomandibular joint, Rhesus monkey

## Abstract

**Background:**

Ankylosis in the temporomandibular joint (TMJ) is known to have various etiologies in veterinary medicine. We observed a case of fibrous ankylosis of the TMJ in a newly imported rhesus monkey (*Macaca mulatta*).

**Case presentation:**

Moderate to severe attrition was found in the middle labial portion of the left maxillary canine. No tenderness around the jaw was detected in the physical examination. The WBC count, CRP level, rheumatoid factor level, and other parameters were normal. Irregularity in the joint surface was observed in both TMJs in the radiographic and computed tomographic examinations, but the left TMJ presented more severe irregularity. It was determined that the removal of the locked portion of the left canine would alleviate the case of lockjaw and allow intubation with an endotracheal tube. Canine tooth crown reduction was performed for both canine teeth. The mouth opening distance slightly (approximately 5 mm) increased up to 20 mm. We concluded that the attrition of canine teeth was not the reason for lockjaw and ankyloses originating from TMJ disease. Fibrotic synovial tissue and joint surface irregularity were observed by necropsy. The presence of fibrocartilage in most areas of the TMJ was confirmed by histology. The diagnosis was fibrous ankylosis of the TMJ associated with DJD.

**Conclusions:**

To the best of our knowledge, this is the first report of degenerative joint disease of the TMJ in a rhesus monkey with fibrous ankylosis of the TMJ.

## Background

Ankylosis of the temporomandibular joint (TMJ) leads to limitations in mouth opening [1] and is one of the most challenging TMJ disorders as it can negatively affect oral-related daily function, such as mastication [2]. Traditionally, the causes of ankylosis are considered either intra-articular (factors within the TMJ; true ankylosis) or extra-articular (factors outside the joint; false ankylosis). Furthermore, true ankylosis cases are considered either bony ankylosis or fibrous ankylosis cases [1]. Consequently, jaw functions such as maximal incisal opening and lateral excursive movements progressively decrease [3]. We found a case of fibrous ankylosis of the TMJ in a newly imported monkey. The purpose of this case presentation is to report fibrous ankylosis of the TMJ with degenerative joint disease (DJD) in a rhesus monkey.

## Case presentation

An 8-year-old male rhesus macaque weighing 7.7 kg was imported from China and was intended to be a recipient of a heterotopic porcine heart transplantation. The animal experiments were approved by the Institutional Animal Care and Use Committee (IACUC) of the Biomedical Research Institute at the Seoul National University Hospital, an AAALAC-accredited facility (IACUC number: 14–0034-C2A0). The monkeys were maintained in single-housed cages and had daily access to a certified primate biscuit diet (2050C, Harlan, Indianapolis, IN, USA) and unlimited access to water. Their room was maintained at 24 ± 4 °C and a relative humidity of 50 ± 10%, with a 12-h artificial light-dark cycle (7:00 am onset) and with 13–18 air changes per hour.

**Fig. 1:**
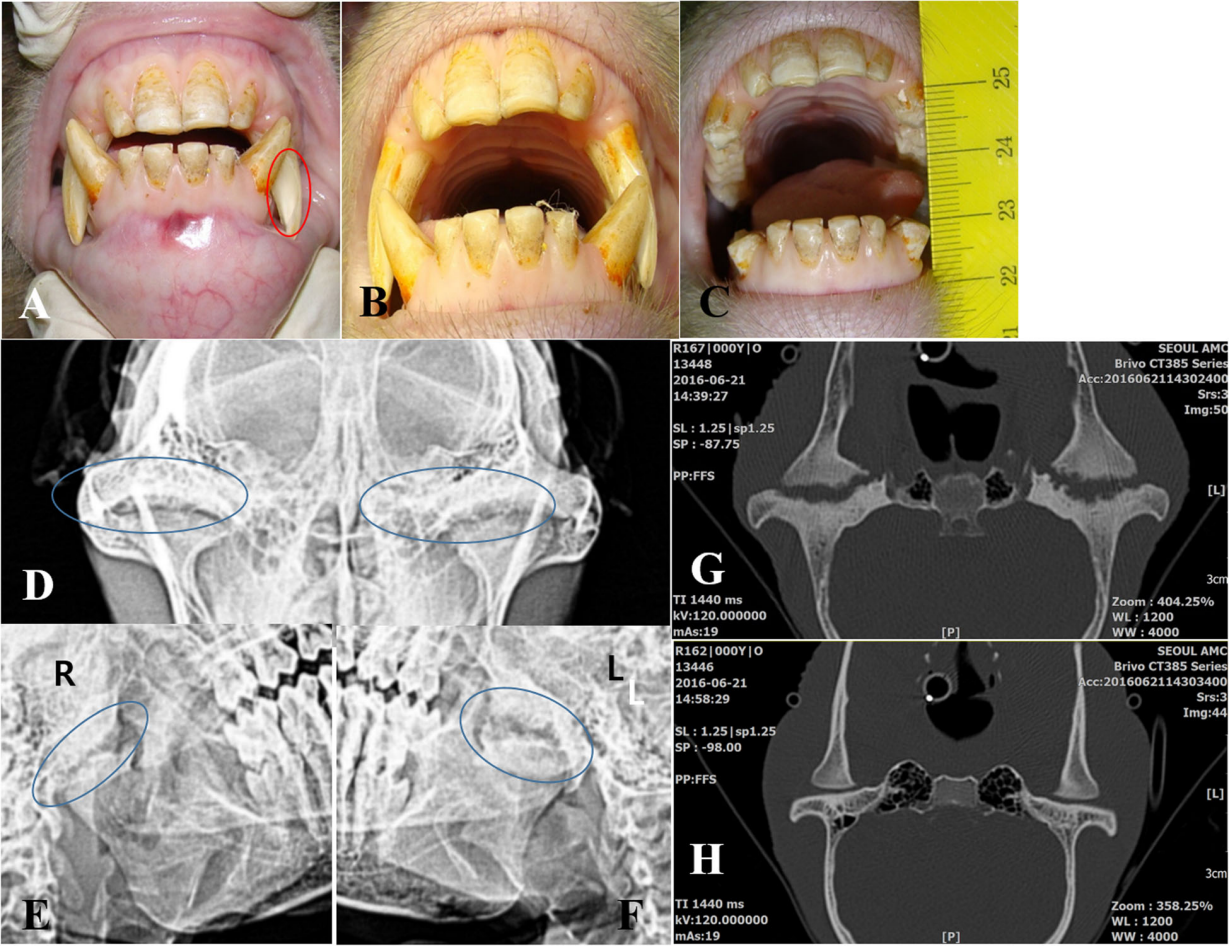
Images of the teeth and radiographic and computed tomographic images of the temporomandibular joint (TMJ). **a** The labial portion of the canine tooth (red ellipse line) in the left maxilla is worn down by the opposite portion in the left mandible. **b** The jaw is maximally opened, but locking occurs between the left canine teeth. **c** After crown reduction of the canine teeth was performed, the jaw could be opened to a greater extent than shown in (**b**). However, normal mouth opening was not performed (maximum 2 cm). **d** The left TMJ shows a more prominent irregular joint surface than does the right TMJ on the anterior-posterior view. **e** & **f** The joint space widening and joint surface irregularity are more severe in the left TMJ than in the right TMJ on the left and right oblique views. The blue ellipse lines indicate the left and right TMJs. **g** The joint space widening and joint surface irregularity are more severe in the left TMJ than in the right TMJ. **h** TMJ image of a normal rhesus monkey

After quarantine, lockjaw was identified (Fig. 1b and supplementary video 1). A supplementary video is available at http://blog.naver.com/jmyw/220665082187. The animal was sedated for physical examination, canine tooth crown reduction, computed tomography (CT), and euthanasia using intravenous medetomidine (0.2 mg/kg, Sedastart, Yuhan, Seoul, Korea) and ketamine (5 mg/kg, Yuhan Ketamine 50 Inj, Yuhan, Seoul, Korea). A physical examination of the head and neck was conducted to identify the etiology of lockjaw in this monkey. We monitored how the rhesus monkey moved food into the oral cavity. Moderate to severe attrition was identified in the middle labial portion of the left maxillary canine, which was caused by the locked portion of the left mandibular canine (Fig. 1a). No tenderness around the jaw was detected in the physical examination. The monkey used its incisor tooth to nibble the food (supplementary video 2).

**Additional file 1.**

**Additional file 2.**

We performed CBC, blood chemistry, radiographic and computed tomographic examinations. For comparison with normal TMJ appearance, we conducted CT examination with the other monkey with a normal TMJ. CT was performed using a multislice scanner (Brivo 380, GE Medical System, Seoul, Korea) under the following conditions: 100 kVp, 10 mAs, 0.625 to 2.5-mm slice thickness, and helical scan type with the animal under general anesthesia with isoflurane. To exclude rheumatoid arthritis, the rheumatoid factor level (by turbidity immunoassay) was analyzed. The WBC count, CRP level, rheumatoid factor level, and other parameters were normal (Table 1). More severe irregularity in the joint surface was observed in the left TMJ compared with the right TMJ in the radiographic and computed tomographic examinations (Fig. 1d-h), and the diagnosis based on these examinations was DJD of both TMJs with fibrous ankylosis.

**Table 1:** Observation periods after canine tooth crown reduction in macaques

Item	Results	Item	Results	Item	Results
WBC	4.71	TP	6.9	TCHO	58
NEU	3.06	ALB	3.8	LDH	425
LYM	1.3	ALP	196	CPK	117
MON	0.2	ALP	627	AMYL	369
EOS	0	AST	44	LIP	18
BAS	0	ALT	18	Ca	7.8
RBC (M/μl)	3.49	BUN	22.3	P	5.1
MCV	86.5	CRE	0.5	NH3	94
HCT (%)	30.2	GLU	74	CRP	0.01
MCH	25.8	TBIL	0.4	rheumatoid factor	< 1.0
MCHC	29.8	DBIL	0.2		
HB (g/dl)	9	GGT	62		
PLT (K/μl)	314	TG	55		

It was determined that the removal of the locked portion of the left canine would alleviate the case of lockjaw and allow intubation with an endotracheal tube. Canine tooth crown reduction was performed for both canine teeth. Briefly, the canine tooth is cut to the level of the incisor teeth using a disc bur. A 1.5-mm-diameter bur removes the pulp and dentin with saline irrigation to prevent thermal injury, and the removed pulp cavity has a larger diameter at the base than at the cutting surface. A cotton pellet soaked with a hemostatic agent (dental formocresol; AGSA JAPAN CO., Osaka, Japan) is used to control the bleeding induced by pulpotomy. A phosphoric acid etchant (CharmEtch 35 HV; DentKist Inc., Gunpo, Gyeonggi-do, Korea) is applied for 20 s for strong adhesion to the filling material. The cavity is washed with a 5.25% sodium hypochlorite solution with antimicrobial effects and then dried gently with air. The cavity is filled with calcium hydroxide/iodoform paste (Vitapex, Neo Dental Clinical Co., Tokyo, Japan) and with glass-ionomer restorative cement (Fuji IX GP; GC Corporation, Tokyo, Japan) according to the manufacturers’ protocols. An antibiotic (cefazolin 20 mg/kg, bid, cefazoline injection 1 g, Chong Kun Dang, Seoul, Korea) and an analgesic (meloxicam 0.1 mg/kg, sid, Metacam 5 mg/ml, Boehringer Ingelheim, Seoul, Korea) are injected intramuscularly for 3 days after canine tooth reduction. The distance of mouth opening was evaluated. The mouth opening distance slightly increased, and the maximum distance of mouth opening was approximately 20 mm after the locked portion of the left canine teeth was removed (Fig. 1c and supplementary videos 3 and 4). We concluded that the attrition of canine teeth was not the reason for the lockjaw and ankyloses originating from TMJ disease.

**Additional file 3.**

**Additional file 4.**

**Fig. 2:**
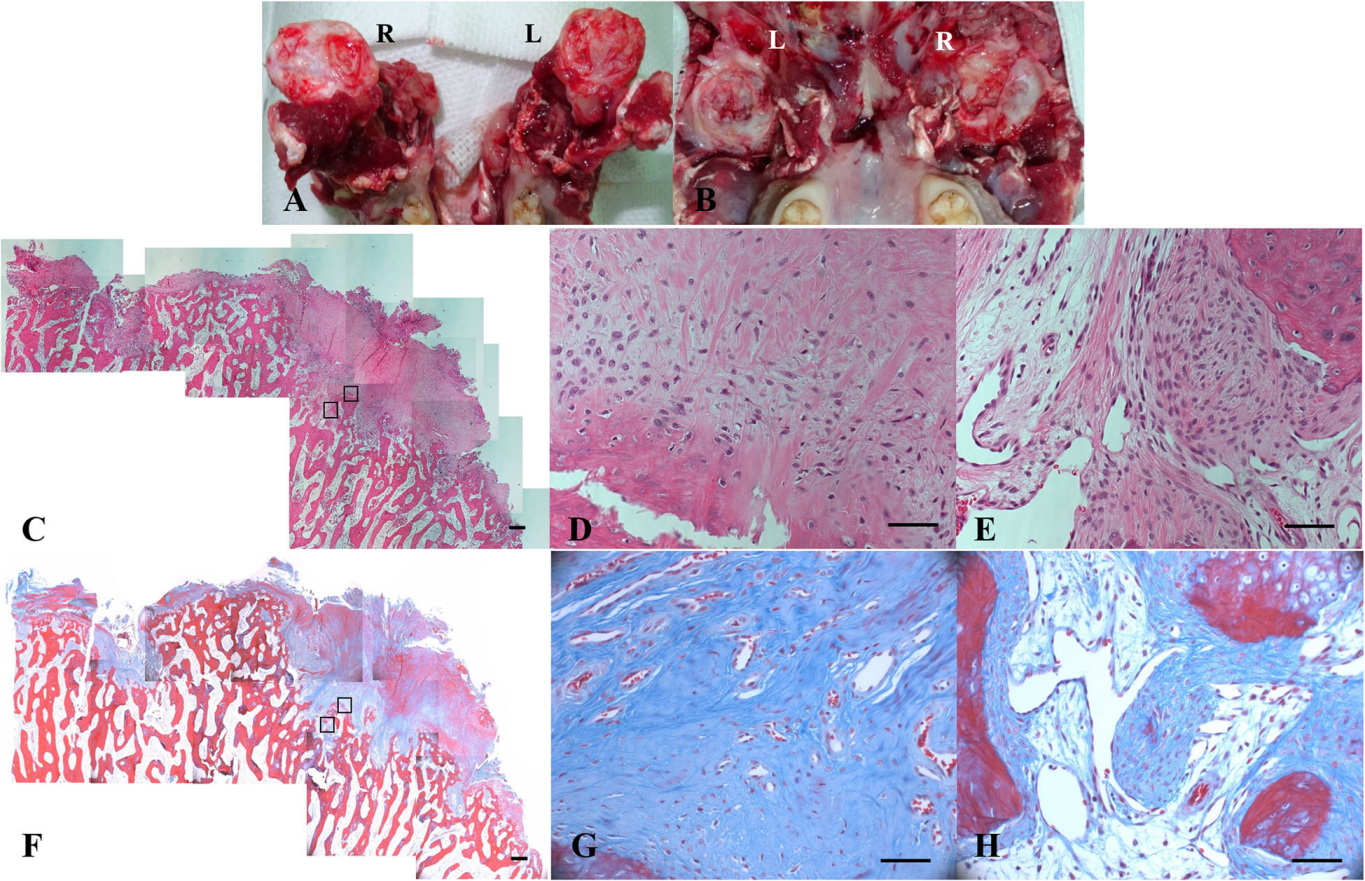
Gross images of the TMJ and microscopic images of the articular surface of the TMJ of the right mandible taken during necropsy. **a** & **b** Degenerated cartilage and adhesive fibrotic tissue are identified in both TMJs (**a**; mandible, **b**; temporal bone). **c** and **f** The articular cartilage of the TMJ is replaced by fibrotic tissue in the scan view. **d** and **g** Magnified image of the right square in (**c)** and (**f**). Fibrocartilage in most areas of the TMJ is identified, and normal cartilage is absent. This fibrocartilage is more cellular than normal articular cartilage, and the unevenly distributed collagen arrays are disorganized. There is no inflammatory reaction. **e** and **h** Magnified image of the left square in (**c**) and (**f**). Proliferative bland-looking fibroblasts are located in marrow tissue and adjacent bony tissue. **c**, **d**, and **e**; **h** & **e** staining. **f**, **g**, and **h** Masson’s trichrome staining. Bar = 50 μm

After the heterotopic porcine heart transplantation experiment was finished, euthanasia was performed by exsanguination under deep anesthesia with thiopental sodium (50 mg/kg, Pentothal sodium injection 0.5 g, JW Joongwae Pham, Seoul, Korea) after sedation. We conducted necropsy and histological examination (H&E and Masson’s trichrome stain) on the TMJ joint. The masseter muscle appeared normal, and fibrotic synovial tissue and joint surface irregularity were observed by necropsy (Fig. 2a&b). The presence of fibrocartilage in most areas of the TMJ was confirmed by histology (Fig. 2c-h). The diagnosis was fibrous ankylosis of the TMJ associated with DJD.

## Discussion and conclusions

The primary etiologies of ankylosis in cats and dogs include trauma and tumors [4]. The etiologies of ankylosis in humans include infection, trauma, dental treatment, TMJ disorders (rheumatoid arthritis, DJD), tumors, oral care, drugs, radiotherapy, chemotherapy, congenital malformations, and miscellaneous disorders [1]. In this case, it appeared that ankylosis of the TMJ emerged as DJD (true ankylosis) radiologically and histologically. Rheumatoid arthritis is a systemic inflammatory condition that affects the TMJ and can cause ankylosis [5]. To exclude rheumatoid arthritis, we determined the level of rheumatoid factor. However, the level of rheumatoid factor was normal, and no inflammatory reactions were found histologically. There was no tenderness, stiff muscles or scar tissue around the TMJ in the physical examination, and there was no elevation of the WBC count or CRP level. These results excluded the etiologies of trauma, muscle contracture, inflammation and infection. There were proliferative bland-looking fibroblasts in marrow tissue and adjacent bony tissue, as shown in Fig. 2e and h. This characteristic implied benign tumor-like morphology of fibroblasts; however, a benign tumor was likely not the etiology because fibrous ankylosis existed on both TMJs. There was no possibility of dental treatment, oral care, drugs, radiotherapy, chemotherapy, or congenital malformations. Taken together, this case of fibrous ankylosis of the TMJ associated with DJD had no definite causes.

One possible cause of DJD in this case is the left canine teeth being locked. Unlike in humans, permanent canine tooth eruption occurs at ages of 46 months and 42 months in the maxilla and mandible, respectively, in rhesus monkeys (*Macaca mulatta*) [6]. Complete maturation of the canine teeth (closed apex) has been confirmed by radiographic assessments of monkeys between 6 and 7 years old [7]. During this period, from when canine teeth erupt to when they are completely mature, male canine teeth become long, sharp, and powerful for survival in the wild. As canine teeth become longer and sharper after eruption, like Dracula teeth, attrition can be induced by the contact of canine teeth. We observed several mild cases of attrition that did not result in locking of the canine teeth where the canine teeth contacted the tip of the dental crown in male rhesus monkeys in our institute. However, there was moderate to severe attrition in the locked portion in the middle labial portion of the left maxilla canine in this case. Locking in the middle portion of the canine teeth rather than in the tip of these teeth might reduce the amount of anterior-to-posterior movement of the mandible necessary for normal mouth opening in this species, making it impossible to open the mouth. The monkey might try repeatedly to open the mouth using large forces, which leads to attrition of the left maxilla canine. This attempt to open the mouth may result in high levels of joint stress and DJD in the TMJ. Temporomandibular disease can occur under occlusal conditions and trauma [8]. Trauma can be subdivided into macrotrauma and microtrauma. Microtrauma, such as bruxism or clenching, refers to any small forces that are repeatedly applied to structures over a period of time. The theory of microtrauma may support this case. Another supportive finding is that the case of DJD was more severe in the left TMJ than in the right TMJ in response to attrition on the left side only. The risk for DJD in humans is higher in those who are overweight, have one leg of a different length and have jobs that result in high levels of joint stress [9].

It is important to take images of good quality of TMJ ankylosis to evaluate the type and extent of the deformity. Computed tomographic images (for determining the diagnosis, prognosis and treatment plan) and magnetic resonance images (for soft tissue examinations) are better than plain radiography for evaluating the TMJ [10]. A limitation in this case report is that magnetic resonance imaging was not performed to evaluate the soft tissue. In conclusion, we described fibrous ankylosis of the TMJ with DJD that had no definite causes in a rhesus monkey. To the best of our knowledge, this is the first report of fibrous ankylosis of the TMJ with DJD in a rhesus macaque.

## Data Availability

Not applicable.

## Abbreviations

AAALAC: Association for Assessment and Accreditation of Laboratory Animal Care
CBC: Complete blood count
CRP: C-reactive protein
DJD: Degenerative joint disease
IACUC: Institutional Animal Care and Use Committee
TMJ: Temporomandibular joint
WBC: White blood cell
